# Comparative Efficacy of Potassium-Competitive Acid Blockers (P-CABs) Versus Proton Pump Inhibitors (PPIs) in the Management of Erosive Esophagitis: A Systematic Review

**DOI:** 10.7759/cureus.96916

**Published:** 2025-11-15

**Authors:** Haseeb Raza, Bhavna Singla, Shivam Singla, Sunita Kumawat, Jeevanjyot S Malhans, Jasleen Kaur, Muhammad Usman, Ishrat Fatima, Rana Salman

**Affiliations:** 1 Pharmacology, Akhtar Saeed Medical and Dental College, Lahore, PAK; 2 Emergency Medicine, Basildon University Hospital, Basildon, GBR; 3 Emergency Medicine, Queen's Medical Centre, Nottingham, GBR; 4 General Surgery, District Headquarters Hospital, Gujranwala, PAK; 5 Internal Medicine, Erie County Medical Center Health Campus, Buffalo, USA; 6 Internal Medicine, TidalHealth Peninsula Regional, Salisbury, USA; 7 Internal Medicine, St. Francis Medical Center, Lynwood, USA; 8 Internal Medicine, Maharishi Markandeshwar Institute of Medical Sciences and Research, Ambala, IND; 9 Internal Medicine, King Edward Medical University, Lahore, PAK; 10 Internal Medicine, Fatima Jinnah Medical University, Lahore, PAK; 11 Internal Medicine, Bahawal Victoria Hospital, Bahawalpur, PAK

**Keywords:** acid suppression, erosive esophagitis, gastroesophageal reflux disease, maintenance therapy, proton pump inhibitors, randomized controlled trials, vonoprazan

## Abstract

This systematic review evaluates the efficacy and safety of vonoprazan and compares it with proton pump inhibitors (PPIs) for the healing and maintenance of erosive esophagitis (EE). A comprehensive search identified five randomized controlled trials published within the last five years, encompassing both short-term healing and long-term maintenance phases. Across studies, vonoprazan consistently demonstrated noninferiority and, in many cases, superiority to lansoprazole, with higher healing rates and lower recurrence, particularly in patients with severe disease (Los Angeles Grade C/D). Long-term data up to five years confirmed sustained efficacy and revealed histopathological changes such as hypergastrinemia and parietal or foveolar hyperplasia with vonoprazan, but no evidence of neoplastic transformation. Additionally, alternate-day dosing showed promising efficacy, suggesting potential cost-effectiveness and mitigation of hypergastrinemia risk. While most evidence originates from Asian populations and some studies had open-label designs, evidence suggests vonoprazan as a potent alternative to PPIs for both induction and maintenance of EE. These results provide clinically meaningful insights for optimizing acid-suppressive therapy and highlight the need for broader, global trials.

## Introduction and background

Erosive esophagitis (EE), a severe manifestation of gastroesophageal reflux disease (GERD), is characterized by mucosal breaks in the esophageal epithelium caused by persistent exposure to gastric acid [1]. It is a condition associated with substantial morbidity, including symptoms of heartburn, regurgitation, dysphagia, and, in advanced cases, complications such as strictures and Barrett’s esophagus. For decades, proton pump inhibitors (PPIs) have been the cornerstone of treatment, providing effective acid suppression and enabling mucosal healing in the majority of patients [2]. However, despite their widespread use, PPIs have important limitations, including a delayed onset of action, inter-individual variability due to CYP2C19 metabolism, meal dependency (requiring activation in an acidic environment and intake before meals), incomplete nocturnal acid suppression, and a gradual reduction in efficacy over time that may mimic clinical “tolerance” due to compensatory hypergastrinemia rather than receptor desensitization. Moreover, a subset of patients experiences partial or non-response to standard PPI therapy, highlighting an unmet clinical need for more potent and reliable therapeutic options [3].

Potassium-competitive acid blockers (P-CABs), such as vonoprazan and tegoprazan, have emerged as promising alternatives to PPIs. Unlike PPIs, which irreversibly inhibit the active form of the H⁺/K⁺-ATPase enzyme after acid stimulation, P-CABs reversibly and directly block the potassium-binding site on the gastric proton pump. This allows P-CABs to achieve immediate, potent, and sustained acid suppression independent of meal timing or proton pump activation [4,5]. Vonoprazan, for instance, reaches maximal plasma concentration within approximately two hours and maintains prolonged inhibitory activity due to its strong, reversible binding and high acid stability. Clinical trials have demonstrated that vonoprazan, the most extensively studied agent in this class, provides faster symptom relief and higher rates of mucosal healing, even in patients with high-grade EE or those with suboptimal responses to PPIs. Long-term studies further suggest that P-CABs may offer more durable maintenance of remission and a favorable safety profile [6,7]. Importantly, their efficacy appears consistent across diverse populations, including Asian cohorts where PPI metabolism and response rates vary due to genetic factors.

Given the growing body of evidence, a systematic review synthesizing the comparative efficacy of P-CABs versus PPIs in EE management is timely and clinically relevant. Such an evaluation will not only clarify the therapeutic value of P-CABs in healing and maintaining remission of EE but also inform future clinical practice guidelines and decision-making. The objective of this review is to comprehensively assess and compare the efficacy and safety of P-CABs versus PPIs in the management of erosive esophagitis, drawing on evidence from randomized controlled trials and long-term clinical studies.

## Review

Materials and methods

Protocol and Guidelines

This systematic review was conducted in strict accordance with the Preferred Reporting Items for Systematic Reviews and Meta-Analyses (PRISMA) guidelines [8], ensuring methodological transparency and reproducibility.

Search Strategy

A comprehensive search was performed in PubMed/MEDLINE, Embase, and the Cochrane Central Register of Controlled Trials (CENTRAL), supplemented by manual reference screening, in accordance with PRISMA guidelines [8]. The search was restricted to the last five years, English language, human studies, and randomized controlled trials (RCTs). Core keywords and controlled vocabulary terms included “erosive esophagitis,” “reflux esophagitis,” “GERD,” “vonoprazan,” “potassium-competitive acid blocker,” “P-CAB,” “proton pump inhibitor,” “PPI,” “lansoprazole,” “esomeprazole,” and “rabeprazole.” Boolean operators and database-specific indexing terms (e.g., Medical Subject Headings (MeSH), Emtree) were used to maximize sensitivity. Duplicates were removed, and all titles and abstracts were independently screened by two reviewers, followed by full-text evaluation to determine eligibility, with disagreements resolved by consensus.

Eligibility Criteria (PICO Framework)

Eligibility criteria were guided by the PICO (Population/Patient/Problem, Intervention, Comparison, and Outcome) framework [9]. The included studies enrolled adult patients (≥18 years) with endoscopically confirmed erosive esophagitis, compared vonoprazan as the intervention with any PPI as the comparator, and reported outcomes such as endoscopic healing, maintenance of remission, recurrence, symptom control, or long-term safety. Exclusion criteria were studies of non-erosive reflux disease, pediatric populations, conference abstracts, protocols, reviews, meta-analyses, and non-comparative designs, as well as trials lacking a direct PPI comparator or clinically relevant outcomes. Data extraction was performed using a standardized template capturing study design, population, interventions, comparators, outcomes, and follow-up. Risk of bias was assessed using the Cochrane Risk of Bias 2 (RoB 2) tool [10]. Author-selected keywords for indexing included vonoprazan, potassium-competitive acid blocker, P-CAB, erosive esophagitis, proton pump inhibitor, PPI, gastroesophageal reflux disease, maintenance therapy, and randomized controlled trials.

Study Selection and Data Extraction

All retrieved articles were independently screened by reviewers based on title and abstract, followed by full-text review of potentially eligible studies. Data extraction was performed using a standardized template that recorded study characteristics, design and setting, sample size, intervention and comparator regimens, duration of follow-up, and key outcomes. Discrepancies were resolved through discussion until consensus was reached.

Risk of Bias Assessment

The Cochrane RoB 2 (RoB 2) tool [10] was applied to assess methodological quality. Domains evaluated included adequacy of the randomization process, deviations from intended interventions, completeness of outcome data, reliability of outcome measurements, and potential selective reporting. Each trial was rated as having low risk, some concerns, or high risk of bias across these domains.

Data Synthesis

Given the heterogeneity in trial design, dosing strategies, and endpoints, a narrative synthesis was undertaken. The evidence was organized to emphasize comparative efficacy of vonoprazan versus PPIs in healing and maintenance of erosive esophagitis, long-term safety findings from extended follow-up studies, and novel dosing strategies such as alternate-day administration.

Results

Study Selection Process

**Figure 1:**
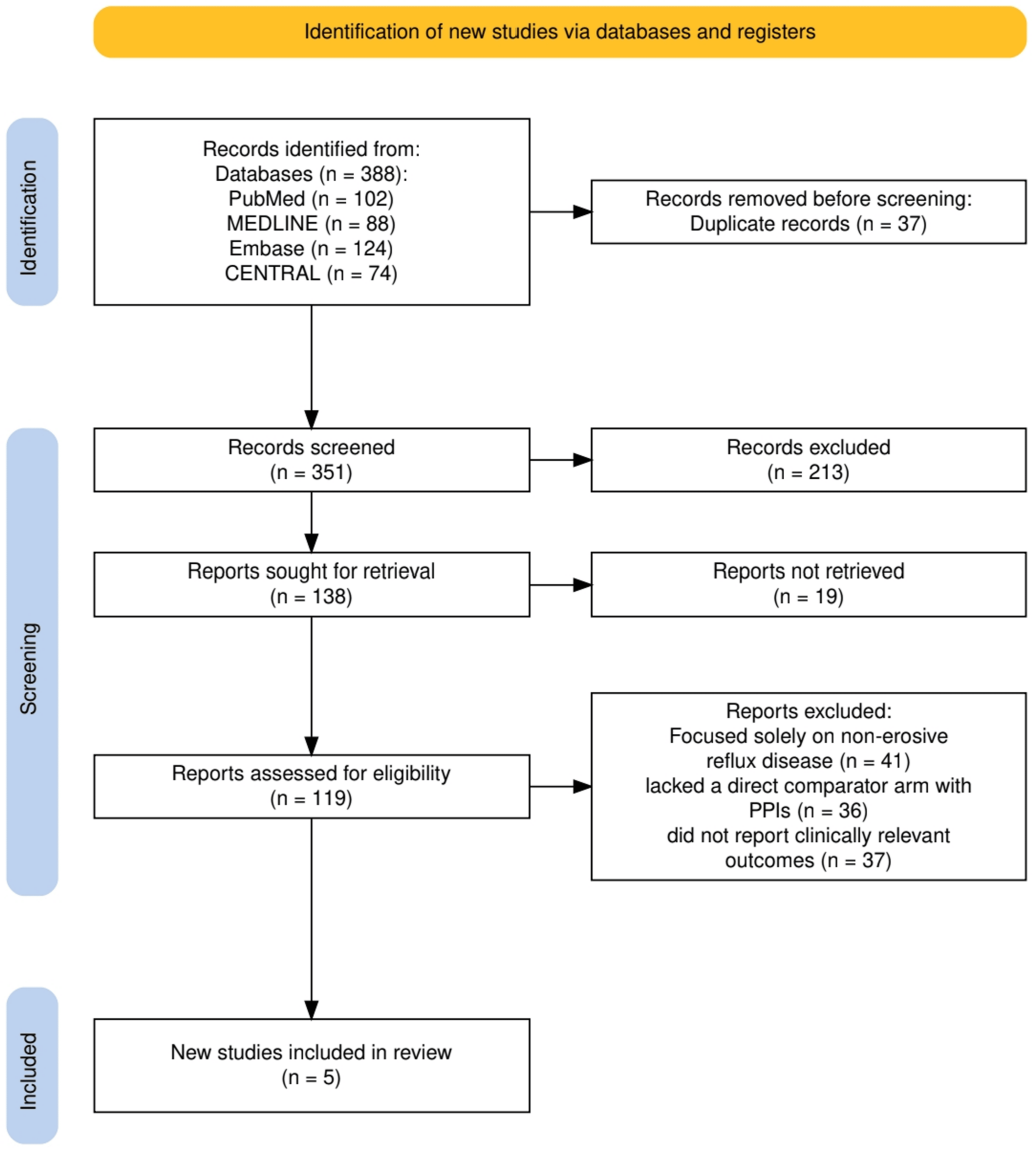
The PRISMA flowchart representing the study selection process. PRISMA: Preferred Reporting Items for Systematic Reviews and Meta-Analyses

The selection process for this review is outlined in Figure 1, which presents the PRISMA flow diagram. A total of 388 records were identified through database searches, including PubMed (n=102), MEDLINE (n=88), Embase (n=124), and CENTRAL (n=74). After removal of 37 duplicate records, 351 unique studies were screened based on titles and abstracts. Of these, 213 studies were excluded for not meeting the inclusion criteria. Full texts were sought for 138 reports, of which 19 could not be retrieved. The remaining 119 full-text articles were assessed for eligibility, and 114 were excluded: 41 focused solely on non-erosive reflux disease, 36 lacked a direct comparator arm with PPIs, and 37 did not report clinically relevant outcomes. Ultimately, five randomized controlled trials met the eligibility criteria and were included in the final systematic review.

Characteristics of the Selected Studies

The characteristics of the selected randomized controlled trials are summarized in Table 1, providing a detailed overview of the study design, populations, interventions, comparators, duration, and outcomes. Collectively, these trials compared vonoprazan with lansoprazole, the standard PPI, in both the induction of healing and the maintenance of remission in erosive esophagitis. The largest study demonstrated that vonoprazan was not only noninferior but also superior in terms of healing and maintaining remission, with the most pronounced benefits observed in patients with severe disease. Long-term evaluations over three and five years confirmed vonoprazan’s ability to maintain remission effectively while highlighting safety signals such as hypergastrinemia and mucosal hyperplasia, though without evidence of neoplastic progression. Trials conducted in broader Asian populations reinforced vonoprazan’s superiority in preventing recurrence compared with lansoprazole, while an innovative alternate-day dosing study revealed that efficacy could be maintained even with reduced dosing frequency, potentially offering cost and safety advantages. Taken together, these studies underscore vonoprazan’s therapeutic potential as a potent and reliable alternative to PPIs in erosive esophagitis management, while also emphasizing the need for ongoing evaluation of long-term safety and applicability to diverse populations.

**Table 1:** Characteristics of randomized controlled trials comparing vonoprazan and proton pump inhibitors in the management of erosive esophagitis. P-CAB: Potassium-competitive acid blocker PPI: Proton pump inhibitor OD: Once daily EE: Erosive esophagitis LA: Los Angeles (classification system for grading severity of erosive esophagitis) C/D: Grade C and Grade D (severe categories in the Los Angeles classification) VPZ: Vonoprazan LZ, LPZ: Lansoprazole RCT: Randomized controlled trial GERD: Gastroesophageal reflux disease QOD: Every other day (from Latin *quaque altera die*) FSSG: Frequency Scale for the Symptoms of Gastroesophageal Reflux Disease GSRS: Gastrointestinal Symptom Rating Scale VISION: Vonoprazan Investigation for Safety and Maintenance in Erosive Esophagitis program, a long-term clinical research initiative evaluating the efficacy and safety of vonoprazan for maintenance therapy in patients with healed erosive esophagitis

Study	Design and Setting	Population (Sample size, Inclusion criteria)	Intervention (P-CAB)	Comparator (PPI)	Duration	Outcomes
Laine et al. (2023) [11]	Multicenter, double-blind, randomized controlled trial	Adults with erosive esophagitis, n=1024 (healing phase), 878 (maintenance)	Vonoprazan 20 mg OD (healing); 10 or 20 mg OD (maintenance)	Lansoprazole 30 mg OD (healing); 15 mg OD (maintenance)	8 weeks (healing); 24 weeks (maintenance)	Vonoprazan noninferior and superior to lansoprazole for healing (92.9% vs 84.6%) and maintenance (10 mg diff 7.2%; 20 mg diff 8.7%). Greatest benefit in severe EE (LA Grade C/D).
Uemura et al. (2025) (VISION, five-year) [12]	Phase IV, randomized, open-label, long-term study (Japan)	Adults with healed EE, n=202 (139 VPZ, 69 LZ)	Vonoprazan (VPZ) 10 mg OD (maintenance, after induction with 20 mg)	Lansoprazole (LPZ) 15 mg OD (maintenance, after induction with 30 mg)	260 weeks (5 years)	No malignant histological changes observed. Higher gastrin levels and parietal/foveolar hyperplasia with vonoprazan vs lansoprazole, but no increased risk of neoplasia. Both maintained EE effectively long term.
Xiao et al. (2024) [13]	Multicenter, double-blind, double-dummy, phase 3 RCT (China, Korea, Malaysia)	Adults with healed EE, n 703	Vonoprazan (VPZ) 10 mg OD or 20 mg OD	Lansoprazole (LZ) 15 mg OD	24 weeks	EE recurrence: VPZ 10 mg (13.3%), VPZ 20 mg (12.3%) vs LZ 25.5%. Both VPZ arms superior to LZ (absolute risk reduction ~12–13%). Well tolerated across groups.
Haruma et al. (2023) (VISION three-year interim) [14]	Phase IV, open-label, randomized, parallel-group trial (Japan)	Adults with healed EE, n=202 (135 VPZ, 67 LZ in maintenance)	Vonoprazan (VPZ) 10 mg OD	Lansoprazole (LZ) 15 mg OD	156 weeks (3 years)	Vonoprazan showed higher rates of parietal/foveolar/G-cell hyperplasia, but no neoplastic changes. Both drugs safe long term; vonoprazan maintained EE effectively.
Matsuda et al. (2022) [15]	Prospective, multicenter, open-label, two-period randomized cross-over study (Japan)	Adults with healed erosive GERD, n=122	Vonoprazan 10 mg every second day	Lansoprazole 15 mg every second day	8 weeks (two 4-week treatment periods)	Vonoprazan QOD superior to lansoprazole QOD for symptom control (93.6% vs 82.1%, p=0.003). Symptom scores (FSSG, GSRS) improved more with vonoprazan, suggesting efficacy even with alternate-day dosing.

Quality Assessment

The quality assessment of the included trials, presented in Table 2, highlights the generally robust design of the evidence base, with several studies demonstrating low overall risk of bias, particularly those employing double-blind, randomized methodologies with objective endoscopic endpoints and prespecified analyses. However, certain limitations were noted in long-term and open-label studies, where performance bias and selective reporting could not be fully excluded. While the five-year and three-year maintenance studies provided invaluable safety insights, their open-label nature and exploratory safety focus introduced some concerns regarding the strength of their conclusions. Similarly, the alternate-day dosing trial, though innovative, relied heavily on subjective symptom reporting, raising the possibility of measurement bias. In contrast, the large multicenter, double-dummy trials conducted across Asia maintained rigorous methodological standards, reinforcing the reliability of their findings. Overall, the evidence can be considered strong and consistent, but cautious interpretation is warranted in the context of open-label and exploratory designs where subtle biases may influence the reported outcomes.

**Table 2:** Risk of bias assessment of randomized controlled trials comparing vonoprazan and proton pump inhibitors in erosive esophagitis. RoB: Risk of bias RCT: Randomized controlled trial ITT: Intention-to-treat FSSG: Frequency Scale for the Symptoms of Gastroesophageal Reflux Disease GSRS: Gastrointestinal Symptom Rating Scale VISION: Vonoprazan Investigation for Safety and Maintenance in Erosive Esophagitis program, a long-term clinical research initiative evaluating the efficacy and safety of vonoprazan for maintenance therapy in patients with healed erosive esophagitis

Study	Randomization process	Deviations from intended interventions	Missing outcome data	Measurement of the outcome	Selection of reported result	Overall RoB judgment
Laine et al. (2023) [11]	Low risk (adequate randomization, double-blind, large sample)	Low risk (double-blind RCT)	Low risk (low attrition, ITT analysis)	Low risk (objective endoscopic endpoints)	Low risk (protocol-driven, prespecified analyses)	Low risk
Uemura et al. (2025) (VISION, 5-year) [12]	Some concerns (open-label, long duration, randomization adequate but 2:1 ratio)	Some concerns (open-label → potential performance bias)	Low risk (long-term follow-up, high retention)	Low risk (histopathology, objective outcomes)	Some concerns (exploratory, safety-focused reporting)	Some concerns
Xiao et al. (2024) [13]	Low risk (multicenter, double-blind, double-dummy, phase 3)	Low risk (blinded, good adherence)	Low risk (large sample, balanced arms)	Low risk (endoscopic confirmation of recurrence)	Low risk (protocol-specified endpoints)	Low risk
Haruma et al. (2023)(VISION 3-year interim) [14]	Some concerns (open-label design)	Some concerns (no blinding → performance bias possible)	Low risk (202 enrolled, retention reported)	Low risk (objective histopathological measures)	Some concerns (interim analysis, selective reporting possible)	Some concerns
Matsuda et al. (2022) [15]	Some concerns (randomized but open-label cross-over)	Some concerns (open-label symptom reporting may bias results)	Low risk (122 enrolled, analysis complete)	Some concerns (symptom-based scales - FSSG, GSRS - subjective)	Low risk (outcomes predefined, statistical testing clear)	Some concerns

Discussion

This systematic review demonstrates that P-CABs, particularly vonoprazan, consistently achieve noninferior and often superior outcomes compared with PPIs in both the healing and long-term maintenance of EE. In Laine et al. [11], vonoprazan showed higher healing rates than lansoprazole at eight weeks (92.9% vs 84.6%) and superior maintenance at 24 weeks, with the greatest benefit observed in patients with severe EE (LA Grade C/D). Xiao et al. [13] confirmed these findings in an Asian population, where recurrence rates at 24 weeks were halved with vonoprazan compared to lansoprazole (12-13% vs 25.5%). Long-term safety data from the VISION (Vonoprazan Investigation for Safety and Maintenance in Erosive Esophagitis) program provide reassurance: Haruma et al. [14] and Uemura et al. [12] reported effective maintenance for three and five years, respectively, with no malignant gastric histology, although vonoprazan was associated with higher gastrin levels and mucosal hyperplasia. Importantly, Matsuda et al. [15] showed that even alternate-day vonoprazan was more effective than alternate-day lansoprazole (93.6% vs 82.1% symptom control, p=0.003), suggesting potential flexibility in dosing strategies. Collectively, the evidence positions vonoprazan as a robust and versatile alternative to PPIs, especially in patients with more severe or refractory disease.

For more than three decades, PPIs have been the gold standard in EE therapy, providing symptomatic relief and promoting mucosal healing [16]. However, their limitations are well established, including delayed onset of action, variability in metabolic response due to CYP2C19 polymorphisms, tachyphylaxis with long-term use, and incomplete nocturnal acid suppression leading to persistent reflux symptoms [17]. Vonoprazan offers pharmacological advantages that directly address these gaps: it achieves rapid, profound, and sustained gastric acid inhibition independent of CYP2C19 metabolism. The results of this review extend and reinforce earlier reports by demonstrating vonoprazan’s superiority over lansoprazole in both acute healing and relapse prevention, with consistent efficacy across multiple Asian cohorts and emerging Western evidence [18]. Importantly, the VISION trials provide the first robust, multi-year safety dataset, showing that although hypergastrinemia and mucosal hyperplasia are more frequent with vonoprazan, these histological changes did not translate into neoplasia over three to five years of follow-up [12,14]. Taken together, these findings not only confirm vonoprazan’s potency but also challenge the long-standing dominance of PPIs, suggesting that P-CABs may be poised to redefine maintenance strategies for EE, particularly in patients with severe disease or incomplete PPI response.

Vonoprazan’s consistent superiority in healing and maintaining remission of severe EE has important clinical implications, as patients with LA Grade C/D disease are often the most challenging to manage with standard PPIs. The rapid and potent acid suppression afforded by vonoprazan could shift treatment paradigms by positioning it as a preferred first-line option in severe EE, potentially reducing relapse, stricture formation, and the need for surgical intervention [19,20]. The VISION trials [12,14] add a nuanced perspective, showing that while vonoprazan is associated with higher rates of hypergastrinemia and mucosal hyperplasia, these histological changes did not translate into neoplastic progression over three to five years, providing reassurance for long-term use in real-world practice [21]. Clinically, these findings suggest that while such changes warrant periodic endoscopic or biochemical monitoring, they are not currently associated with adverse clinical outcomes in the available long-term data. Nevertheless, the predominance of Asian trial populations raises questions about generalizability, as genetic, dietary, and environmental differences may influence both drug metabolism and disease expression; Western studies will be critical before widespread adoption. Equally noteworthy is Matsuda’s demonstration [15] that alternate-day vonoprazan dosing remains effective, which not only opens the door to cost-reduction strategies but may also mitigate hypergastrinemia, a finding that could redefine maintenance regimens if validated in larger cohorts.

While the included studies represent rigorous RCTs, several limitations must be acknowledged. Many of these trials were industry-sponsored, which introduces the possibility of publication and reporting bias, as industry involvement may influence study design, analysis, or selective outcome reporting. The evidence base is also geographically narrow, with most trials conducted in Japan and East Asia, limiting extrapolation to diverse global populations. Although the VISION program provides valuable long-term safety data up to five years, the endpoints remain focused primarily on gastric histology rather than clinically relevant outcomes such as progression to Barrett’s esophagus, development of strictures, or long-term quality-of-life measures. Moreover, comparative trials have predominantly used lansoprazole as the reference PPI, leaving uncertainty about how vonoprazan performs against more potent third-generation PPIs like esomeprazole or rabeprazole. These methodological gaps temper the strength of the conclusions and highlight the need for broader confirmatory studies. Additionally, due to heterogeneity among trial designs, dosing strategies, and endpoints, we did not perform a quantitative meta-analysis, which may have limited the precision of pooled effect estimates. Future large-scale, multinational randomized trials with standardized outcome measures are warranted to confirm and extend these findings.

In clinical practice, vonoprazan holds promise across several therapeutic niches. For patients with severe EE, it could emerge as a first-line option due to its demonstrated superiority in mucosal healing [22]. In cases of incomplete response to PPIs, vonoprazan provides a rational second-line alternative, especially in those with nocturnal symptoms or rapid relapse. Furthermore, PPI-refractory GERD - a significant clinical challenge - may be a domain where vonoprazan offers consistent efficacy, as suggested by its pharmacologic profile. However, barriers remain: cost considerations, drug availability, and patient access differ across health systems, and these factors will influence whether vonoprazan can truly displace PPIs as a frontline therapy [23]. Patient preference, particularly for less frequent dosing regimens such as alternate-day therapy, may also play a role in adherence and long-term satisfaction.

Future research should address several pressing gaps. Trials conducted outside of Asia are urgently needed to validate efficacy and safety in Western populations, where genetic and environmental factors differ substantially. Head-to-head comparisons against newer-generation PPIs like esomeprazole and rabeprazole would help determine whether vonoprazan offers incremental benefit over the best available PPI options, rather than older comparators like lansoprazole [24]. Long-term follow-up beyond five years is essential, especially to monitor for hypergastrinemia-related complications and gastric cancer risk, outcomes that cannot be fully assessed in shorter studies. In addition, dedicated studies in special populations - patients with Barrett’s esophagus, extra-esophageal reflux syndromes, obesity, or other high-risk comorbidities - will be critical to delineate the full therapeutic spectrum of vonoprazan. Such trials will inform whether P-CABs remain a niche therapy for severe EE or evolve into a new standard of care across reflux disease phenotypes.

## Conclusions

This systematic review underscores that vonoprazan, a P-CAB, is not only noninferior but frequently superior to PPIs in both the healing and long-term maintenance of EE, with particular benefits in patients with severe disease. Evidence from multicenter RCTs confirms its faster onset and more consistent acid suppression, while long-term data from the VISION program provide reassurance regarding safety, despite the presence of hypergastrinemia and mucosal hyperplasia without malignant transformation. Importantly, alternative dosing strategies, such as every-other-day administration, suggest opportunities to optimize cost and safety without compromising efficacy. The significance of this study lies in its synthesis of emerging global data, providing critical insights that challenge the long-standing dominance of PPIs and positioning vonoprazan as a potential new standard in the management of EE. By consolidating short-term efficacy, long-term safety, and novel dosing strategies, this review highlights the clinical and scientific importance of P-CABs and sets the stage for future research to expand their role in GERD management.
